# Semiautomated Alignment of High-Throughput Metabolite Profiles with Chemometric Tools

**DOI:** 10.1155/2017/9402045

**Published:** 2017-01-12

**Authors:** Ze-ying Wu, Zhong-da Zeng, Zi-dan Xiao, Daniel Kam-Wah Mok, Yi-zeng Liang, Foo-tim Chau, Hoi-yan Chan

**Affiliations:** ^1^School of Mathematics, Physics and Chemical Engineering, Changzhou Institute of Technology, Changzhou 213002, China; ^2^State Key Testing Laboratory of Food Contact Materials, Changzhou Entry-Exit Inspection and Quarantine Bureau, Changzhou 213002, China; ^3^Chemometrics and Herbal Medicine Laboratory, Department of Applied Biology and Chemical Technology, The Hong Kong Polytechnic University, Hung Hom, Kowloon, Hong Kong; ^4^Dalian ChemDataSolution Technology Co. Ltd., High-Tech Zone, Dalian, Liaoning 116023, China; ^5^School of Chemical and Biological Engineering, Changsha University of Science & Technology, Changsha 410114, China; ^6^State Key Laboratory of Chinese Medicine and Molecular Pharmacology, Shenzhen 518057, China; ^7^Research Center of Modernization of Chinese Medicines, College of Chemistry and Chemical Engineering, Central South University, Changsha 410083, China

## Abstract

The rapid increase in the use of metabolite profiling/fingerprinting techniques to resolve complicated issues in metabolomics has stimulated demand for data processing techniques, such as alignment, to extract detailed information. In this study, a new and automated method was developed to correct the retention time shift of high-dimensional and high-throughput data sets. Information from the target chromatographic profiles was used to determine the standard profile as a reference for alignment. A novel, piecewise data partition strategy was applied for the determination of the target components in the standard profile as markers for alignment. An automated target search (ATS) method was proposed to find the exact retention times of the selected targets in other profiles for alignment. The linear interpolation technique (LIT) was employed to align the profiles prior to pattern recognition, comprehensive comparison analysis, and other data processing steps. In total, 94 metabolite profiles of ginseng were studied, including the most volatile secondary metabolites. The method used in this article could be an essential step in the extraction of information from high-throughput data acquired in the study of systems biology, metabolomics, and biomarker discovery.

## 1. Introduction

Hyphenated chromatographic instruments, coupled to mass spectrometers, have been preferred tools and play a substantial role in the study of complicated problems in systems biology, such as functional genomics, proteomics, and metabolomics, all of which have a large number of targets (genes, proteins, and small molecules) being detected [1–3]. For example, gas or liquid chromatography coupled with mass spectroscopy (GC-MS or LC-MS^*n*^) is widely used to analyze biosamples, such as serum, urine, stool, and cerebrospinal and synovial fluids [3–6]. These analyses are a necessary part of investigating complex systems and help us understand the mechanisms of important life processes.

High-dimensional chromatographic profiles are commonly complicated and consist of thousands of components. The pretreatment of high-throughput data obtained from autosampling instruments is important because it can substantially improve the amount of information extracted from data with numerous experimental variations. The requirements of rapid, accurate, automated, and high-throughput analyses are a challenge to many analytical chemists and biologists who analyze their data conventionally [7]. To chemometricians, major data processing of metabolite profiles includes spectral filtering/smoothing, peak detection, normalization, discrimination analysis, deconvolution, and alignment, among others steps [8, 9]. The retention time shift is very important in profile evaluations and pattern recognition in the study of gene and protein functions, drug toxicology, and metabolomics and especially in biomarker/biomarker pattern discovery using multivariate statistical methods, such as PCA, HCA, and PLS-LDA [10, 11]. The retention time shift strongly contributes to obtaining accurate qualitative and quantitative information on components that are hidden in complicated chromatographic peak clusters by deconvolution methods, which assumes trilinearity in tensor data sets [12]. In general, data treatment, such as correction of the retention time shift, is needed to improve data quality, and it is helpful in obtaining fruitful conclusions in most metabolomics studies.

Recently, many effective strategies have been proposed to align metabolite profiles or fingerprinting. An often-used technique consists of adding internal standards to the samples of interest prior to analysis [13]. The peaks corresponding to the standards are used as references to correct the retention time shifts in the chromatograms. Another method is based on chemometric deconvolution and the comparison of the spectra of a few reference components found in the data. For example, the local least-squares regression model (LLS) was developed on the basis of spectral correlative chromatography (SCC) to identify the presence of selected peaks, which were used to calibrate the retention times obtained in different runs [14, 15]. Essentially, SCC is an approach based on searching for similarities among selected components of spectra. In another study, deconvolution methods such as heuristic evolving latent projections (HELP) and subwindow factor analysis (SFA) were utilized to extract the pure spectra of components and were used as references for alignment [16]. Clearly, the reference component has a significant effect on the alignment results. If successful, the linear interpolation technique (LIT) can be utilized to eliminate the retention time shift. However, these methods have many drawbacks when used to analyze high-throughput profiles or fingerprinting. For example, the reference components must be selected manually. Correlation optimized warping (COW) and its improved versions were selected as alternative methods for both single- and multiple-channel chromatographic data alignment via a piecewise linear stretching and compression strategy in the time axis of the profiles [17, 18]. A long computation time is the primary restriction for the application of this method to high-throughput data. Many commercial metabolomics software or programs, including MetAlign [19], MSFACTs [20], MZmine [21], and XCMS [8, 9], have the capability of retention time alignment for chromatograms; however, all are restricted to a correct mass spectral format. Some of the programs have a maximum capability of 1000 profiles, and the algorithms used are usually proprietary.

In this study, an automated method is proposed to align high-throughput metabolite profiles using the orthogonal projection technique, which is a powerful chemometric strategy [14]. Information from the acquired profiles was used to select a standard profile from the data set, and the standard profile was used as a reference for alignment [22]. Automated target search (ATS), based primarily on the orthogonal projection technique to obtain the elution windows and the exact profile peak of reference components through deduction of the common spectral information, was developed in this study. The determination of the reference components in the standard profile was successfully accomplished by ATS. LIT was utilized for the final correction of retention time shifts. The comprehensive and piecewise partition strategy of the profile guaranteed the proper alignment in the data.

The proposed method was applied to align the GC-MS profile of ginseng acquired under the autosampling high-throughput model. Ginseng, a well-known herb, is widely used to improve psychological and immune system functions, lower blood sugar and cholesterol levels, protect against stress, enhance strength, and promote relaxation [23, 24]. The secondary metabolites in ginseng include terpenoids, alkaloids, polyphenols, and polyketides that have been proven to exhibit certain bioactivities [23–25]. The study of plant metabolite profiles is currently an area of interest in metabolomics and systems biology. It has sufficient complexity to challenge the potentials of the proposed methods and further deliver the principle and operational process of the method.

## 2. Materials and Methods

### 2.1. Automated Target Search (ATS) Method

In this study, the metabolite profile used as a reference for alignment is referred to as the standard profile, whereas the other samples are referred to as sample profiles.

#### 2.1.1. Determination of Representative Data as Reference for Alignment

After obtaining the raw instrumental data with inclusion of chemical features, determination of samples for reference and alignment is the first step. It has no difficulty to understand the reasonability using information content (IC) of the total ion chromatograms (TIC) as a quantitative index. It was calculated by the following equation. All the symbols and their annotations were summarized in Symbols and Annotations.

(1) $$\begin{array}{c} \varphi_{i}=-\sum \left(\;\frac{x_{i}}{\sum x_{i}}\;\right)\log\left(\;\frac{x_{i}}{\sum x_{i}}\;\right), \end{array}$$

where *φ*_*i*_ denotes the IC and *x*_*i*_ refers to the *i*th TIC metabolite profile. IC is a quality indicator and an evaluation of the complexity of the profile; hence, it provides a suitable objective measure to identify the standard profile. According to (1), the profile with a maximum *φ* was selected as the standard profile; however, if a fixed metabolite profile is known and defined as the standard profile, the previous step can be omitted. For example, all other sample profiles must be aligned to one certain profile because a reference is needed to achieve the experimental objective.

#### 2.1.2. Data Partition and Comparison for Standard and Aligned Profiling

**Figure 1:**
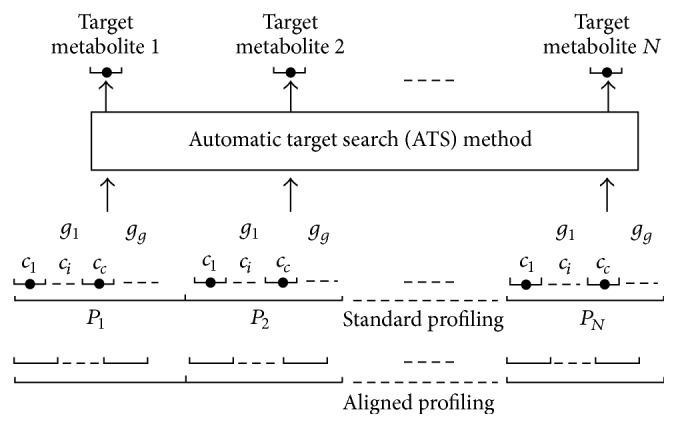
Partition of the standard profile using the ATS method and an illustration of how to find the target components as a reference for alignment.

After determination of standard profile, the left samples/profiles can then be corrected to this reference according to the following procedures. As shown in Figure 1, the standard profile was uniformly partitioned into* N* parts, which depended on the complexity of the profile and the requirements of alignment, and further, each part is divided into *g*_*g*_ peak groups. The numbers* N* and *g*_*g*_ are determined by the complexity of chromatographic profiles and, respectively, suggested to 10–15 and 3–5 in most cases. The first *c*_*c*_ components with the maximum response in* N* partitions were extracted across a threshold of retention time points, such as 50 in this study, and they were further identified as potential reference components. If the target component in certain partition is found in the aligned profile, the corresponding peak location information is recorded and moved into the next partition. Otherwise, the next *g*_*i*_ peak cluster with maximum response is extracted for new computation until the *g*_*g*_ clusters are accomplished, according to the processing sequence with remaining maximum peak response.

**Figure 2:**
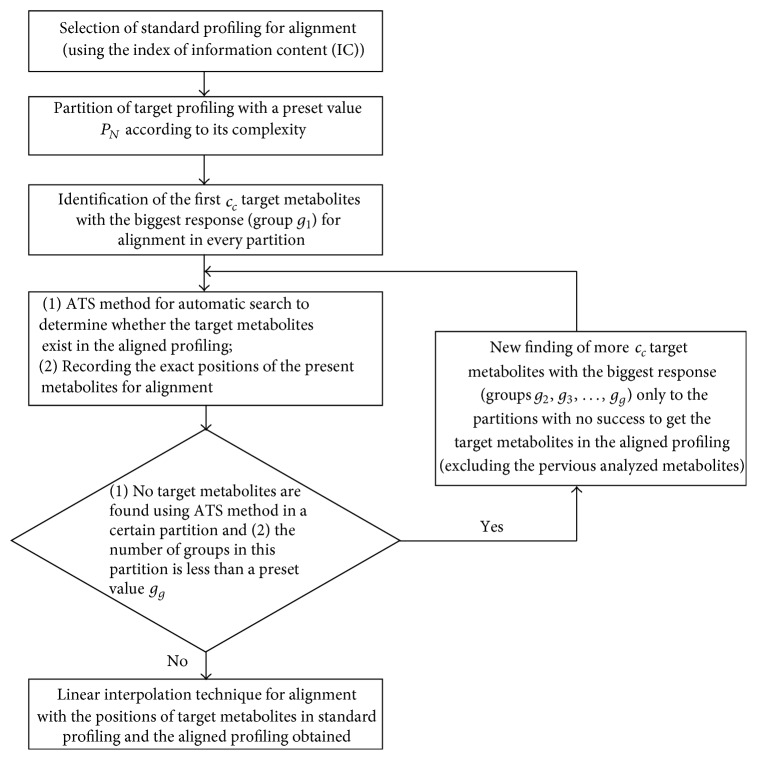
The entire working procedure for the developed method in this study.

The working procedure for profile partitions is illustrated in Figure 1. The exact elution locations of the target components as a reference for alignment between the standard and profiles for alignment are also displayed. As described in the previous section, the first* c* components with a maximum response in each of the* N* uniform partitions of the standard profile were studied using the ATS method. If more than one target component was found under the preset threshold conditions, the one with a minimum value of *ζ* was then selected as a reference in this partition. The exact elution retention time was recorded and used to perform the correction of shift of retention time in the next step. If no component was discovered using the ATS parameters, the partitions were searched for the next *c* maximum components until the information of the targets in the profiles for alignment was acquired or until the prearranged number of component groups *g*_*g*_ for treatment was obtained, or no target components can be found in a certain partition completely. Finally, the target components 1, 2,…, and* N*, all presented in both the standard and profiles for alignment, were utilized to complete the shift correction. In some cases, the ATS failed to find the exact chromatographic peaks of the target components but instead found other locations of the chromatographic profile. Identification of the maximum by searching the closest components of the profile was straightforward.  Figure 2 shows the entire alignment procedure with a clear flowchart. The widely used LIT technique was the last step to record retention time locations of target components for reference. Gong et al. described that linear interpolation is conducted on the total ion chromatograms for rapid calculations or on every mass chromatogram of the entire GC-MS profile to reconstruct the coupled data [16]. All of the operations in the ATS and the profile partition could automatically be implemented with several thresholds. Such a strategy could be effective for processing high-throughput profiles acquired in the study of metabolomics.

#### 2.1.3. Theory for Finding of Component Correlation as Standards for Alignment

In terms of the method principle introduced above, the key issue was how to obtain accurate retention times of the target components in the sample profiles; after the retention times were available, the shift could be corrected using the LIT technique. If one of the target components existed in the data matrix** X** window that was extracted from the standard profile, the ATS method could be used to find the time at which the same component was eluted in the sample profiles (referred to as data set **Y**_*m*×*n*_).

Then, the next procedure introduces the steps about how to find the correlation of target components existing in standard and real data. First, singular value decomposition (SVD) analysis of data** X** was applied to extract component features, a part of the standard profile that includes the possible target components:

(2) $$\begin{array}{c} X\;\;=\;\;USV^{T}. \end{array}$$

Here, orthogonal matrices** U** and** V** are referred to as the score and loading matrices, respectively, and** S** is a diagonal matrix that collects the square root of all of the eigenvalues of data set** X**. The superscript T denotes the transposition of a vector/matrix. In addition, matrix** V** represents all of the spectral information of the included components. If the targeted component contributes to the mixture spectra, its spectrum (vector) would be contained in the hyperplane **V**_*p*_ defined by the first *p* eigenvectors of the abstract matrix spectral** V**. Thus, if a spectral component is present in data matrix** X**, then it belongs to the spectral space spanned by the vectors in **V**_*p*_. Therefore, an orthogonal projection matrix **O**_*P*_ can be defined to identify any spectra in the corresponding data set** Y** of the sample profiles that are related to those in** X**. The presence of the target components can then be determined and used for alignment.

(3) $$\begin{array}{c} O_{P}=\left(\;I-V_{p}{V_{p}}^{T}\;\right). \end{array}$$

The unit matrix** I** (see (3)) has the same dimension as the matrix (**V**_*p*_**V**_*p*_^T^). The matrix **O**_*P*_ can be constructed as follows to avoid the determination of an arbitrary parameter *p*:

(4) $$\begin{array}{c} O_{P}\;\;=\;\;\left(\;I-XX^{+}\;\right), \end{array}$$

where the superscript “+” denotes the generalized inverse of a matrix. Subsequently, every column vector of** Y** and **y**_*i*_ (*i* = 1,…, *m*), which is the spectrum obtained from the *i*th chromatographic point *i*, is projected onto the operator **O**_*P*_ according to the following equation:

(5) $$\begin{array}{c} {y_{i}}^{resi}\;\;=\;\;O_{P}y_{i\;\;}=\;\;\left(\;I-V_{p}{V_{p}}^{T}\;\right)y_{i}\;\left(i=1,\ldots ,m\right). \end{array}$$

The residual vector **y**_*i*_^resi^ is the remaining information after** Y** at the *i*th chromatographic point projecting to** X**. When the spectral space of the abstract matrix **V**_*p*_ equals or includes **y**_*i*_, the Euclidean norm of **y**_*i*_^resi^  (‖**y**_*i*_^resi^‖^2^) becomes zero, except for the presence of noise, because all spectral features related to the reference components in the data are removed through the orthogonal projection operation, as shown in (6); otherwise, the value of ‖**y**_*i*_^resi^‖^2^ will be significantly greater than zero.

(6) yiresi2yiresiTyiresi=OPyiTOPyi=yiTOPTOPyi=0i=1,…,m.

To eliminate the effects of heteroscedastic noises in data sets obtained from actual applications, the congruence coefficient (inner product) *ζ*_*i*_ between the original spectrum **y**_*i*_ and the projected residual vector **y**_*i*_^resi^, as written in (7), is determined as the final index to evaluate the presence of the reference components in the profiles for alignment.

(7) $$\begin{array}{c} \zeta_{i}=\frac{{y_{i}}^{T}{y_{i}}^{resi}}{\left\|{y_{i}}^{T}\right\|\left\|{y_{i}}^{resi}\right\|}\;\left(i=1,\ldots ,m\right). \end{array}$$

After all of the *m* chromatographic scan points (*i* = 1,…, *m*) have been analyzed using ATS, a curve of *ζ*_*i*_ is obtained (see (8)) and the exact locations of the reference component can be obtained from the minimum values in the curve.

(8) $$\begin{array}{c} \zeta_{i}=\left[\;\zeta_{1}\zeta_{2}\cdots \zeta_{m}\;\right]\;\left(i=1,\ldots ,m\right). \end{array}$$

By definition, *ζ* ranges between 0 and 1. A lower value of *ζ*_*i*_ indicates a greater correlation between the spectral features of the targeted components and the corresponding spectrum in the data and, thus, indicates a greater possibility of the targeted components existing at that point. In general, a threshold of 0.1 was used to identify the presence of targeted components in this study through consideration of the background of the coupled chromatographic data. The threshold of the data sets depended on the level of background and noise as well as on the length of each extracted chromatogram that included the target components as a reference. If the background could be reduced using an automatic algorithm, the performance of ATS could be improved. The results could be approximately determined through preanalysis of the example data of the metabolite profile. Because the profile was important for alignment, it was most effective to discover the target components through the use of ATS with preset defaults. It was possible to confuse the target component and its neighboring components if the window size for extraction of the coupled chromatograms was too large and if the overlapping components had a high intensity. However, the constraints of a conservative **ζ** value and several continuous thresholds helped avoid such situations. This effect did not appear to influence the results of the present study.

### 2.2. Data Sets

In this study, the volatile secondary metabolites of ginseng were investigated to demonstrate how the ATS method and data partition strategy work. The retention time shift is an important part of chemical analysis, which is the basis for quality control, plant metabolomics research, absorption, distribution, metabolism, and in vivo toxicity (ADME/TOX) of ginseng.

#### 2.2.1. Sample Extraction

The ginseng samples were ground and crushed. Hexane was added to the samples, and an ultrasonic extraction was performed for 1 hour at room temperature. After centrifugation, the supernatant was analyzed by GC-MS.

### 2.3. Instruments/Analytical Conditions

A GC-MS analysis of the volatile constituents of ginseng was performed on a Shimadzu QP-2010 GC-MS spectrometer (Tokyo, Japan). A DB-5MS capillary GC column (30 m × 0.25 mm, 0.25 *μ*m, Agilent, Santa Clara, USA) was used for the separation. The column temperature was initially set at 100°C, which was increased to 170°C at a rate of 1.5°C/min. The temperature was subsequently increased to 190°C at a rate of 8.0°C/min and finally increased to 240°C at a rate of 2.0°C/min. The inlet temperature was maintained at 270°C. The carrier gas was set to a constant flow of 1.3 mL/min, with a split ratio of 2 : 1. A full-scan mode was used to record the mass spectra of the mass-to-charge ratio (*M*/*Z*) range of 1–380 with a sampling rate of 2 s. The temperatures for the EI-ionization source and interface were set at 200°C and 250°C, respectively.

## 3. Results and Discussion

### 3.1. Implementation

All computer programs used in this study were coded in MATLAB 6.5.0, and all computations were performed on an Intel (R) Core (TM) 2 CPU 6300 (1.86 GHz and 1.87 GHz) with 2 GB of RAM.

### 3.2. Results

**Figure 3:**
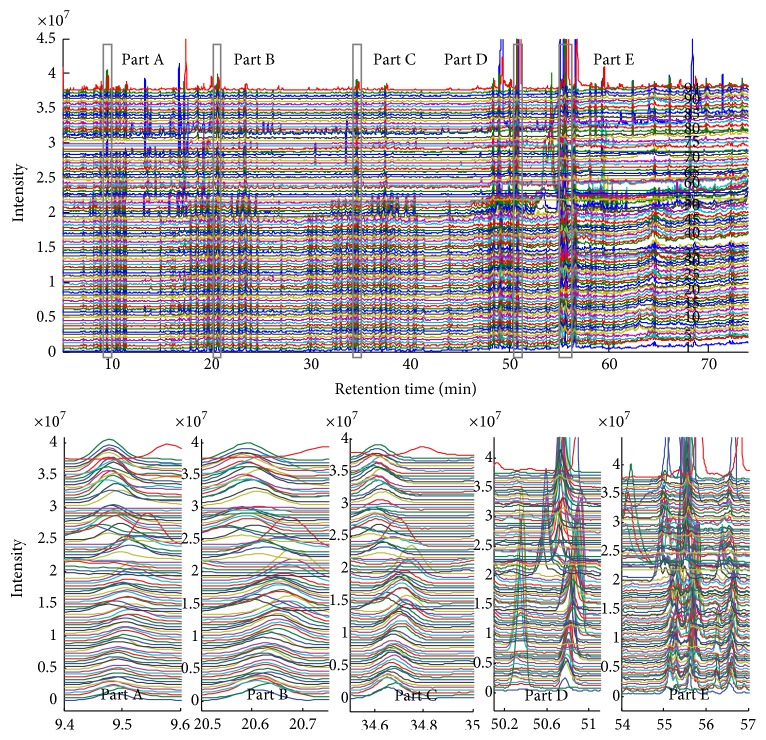
The 94 original metabolite profiles (TIC profiles of GC-MS data) of ginseng before alignment. The five figures from part A to part E correspond to the five elution windows and show the detailed characteristics.

In total, 94 TIC profiles of ginseng were acquired by the GC-MS instrument (Figure 3). The retention time shifts of these profiles were easily observed by visual inspection. The figures (magnified areas of part A to part E in Figure 3) clearly demonstrated the need for alignment before pattern recognition and/or other data processing operations to obtain detailed information embedded in the plant profile. Different profiles exhibited different time shifts between the first and last elution windows. The results for the clustering analysis using PCA or other methods were unacceptable, even for profiles with very similar chemical components. Thus, a two-dimensional retention time shift correction was necessary prior to data evaluation and information extraction. The IC (*φ*) of all 94 TIC profiles obtained from the hyphenated GC-MS were calculated using (1) to determine the standard profile. The first profile in Figure 3 with maximum value of IC was selected as the standard for alignment of the sample profiles. The exact corresponding chromatographic points of the target components were automatically determined and recorded using the ATS to correct the shift of the aligned profiles.

**Figure 4:**
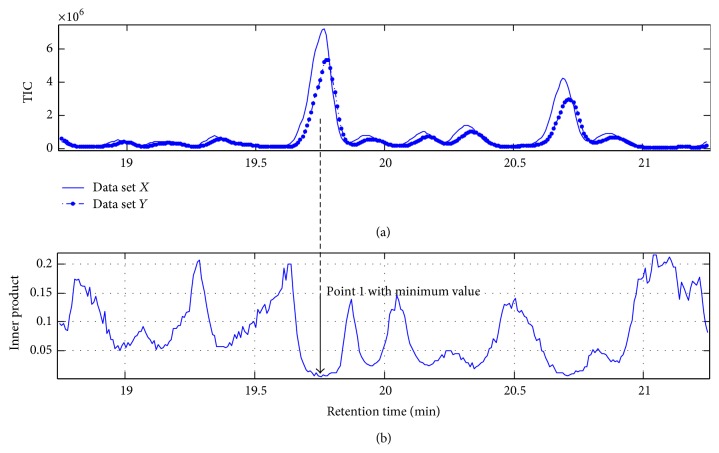
An example of how to obtain the exact retention time information of the target component using the ATS method for alignment. (a) Total ion chromatograms of ginseng data sets** X** and** Y** as examples; (b) the residual graphs of data set** X** projected onto** Y**. Point 1 shows the retention time position with the minimum value of the inner product corresponding to the target component.

Figure 4 shows an example of the retention times of the selected targets in a sample profile determined using the ATS method. Figure 4(a) shows the TIC chromatograms of the standard and a sample profile labeled as data sets** X** and** Y**, respectively. The purpose of ATS was to determine whether the selected target components were present in** Y** and to determine the retention time shift using the **ζ** values as the index. The curve of inner product **ζ** in Figure 4(b) was observed in the same chromatographic elution window shown in Figure 4(a). It was obtained by projecting data set** Y** into the spectral space of** X** with the help of the ATS method. This curve showed that the target components included in data set** X** existed in** Y**; the arrowhead shows the minimum **ζ**, which indicates the accurate retention time of the selected targets. The **ζ** value at this location approached zero, and it was sufficiently small to prove the mutual presence in the standard and the sample profiles. Notably, this was only an example to demonstrate the strategy because the data size of** X** was very small (approximately 50 data chromatographic points) to reduce the interference of neighboring components.

**Figure 5:**
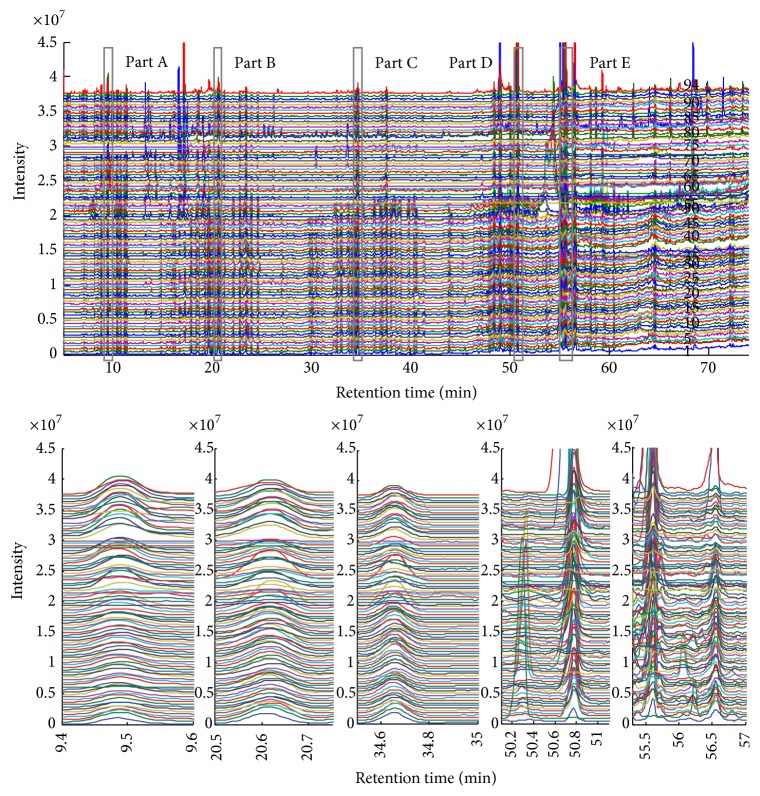
The alignment results of the 94 profiles shown in Figure 4 using the developed method. The five figures from part A to part E correspond to the five elution windows, which are provided to demonstrate the detailed performance.

In this study, we found that 12 partitions (*N*) were sufficient to achieve good performance using the proposed method. The number of component groups (*g*) was not the same for different samples. Only one component selection could determine the target as a reference for alignment, which matched the preset thresholds/parameters. To determine the number of principal factors needed to construct the orthogonal projection matrix **O**_*P*_ defined in the ATS method, the default value was set to 4 or 5 to obtain all the possible spectral attributes included in data set** X**; it should be adjusted according to the complexity of the extracted chromatographic profile. Commonly, a relative large value parameter *p* is necessary because of the absolute inclusion of background and noise in data set** X**. The elution information of the target components was recorded and applied to correct the shift of each sample profile using the LIT technique.  Figure 5 shows the results of the standard and sample profiles as the entire picture and enlarged figures corresponding to the same regions given in Figure 3.  Figure 5 is provided as examples to avoid confusion from the large metabolite profile. The performance of the shift correction was good enough to be delivered to the next step for data processing. The minimum, mean, and maximum correlation coefficients between the mean profile and all 94 independent profiles were improved from 0.1878 to 0.4481, 0.7207 to 0.8122, and 0.8972 to 0.9671, respectively. The final similarities were not significant because of the compositional difference among the profiles and the lack of background correction. The direct alignment of the retention time shift with both signal and background was more convenient and easily attained from the automated method. These aligned profiles could be used for subsequent data analysis in the studies of metabolomics.

### 3.3. Discussion

The ATS method has the capabilities of global search to find selected components from the experimental profile for correction. It comprehensively considers whether the target components used to perform the alignment are present in the regions of interest. If necessary, the whole GC-MS profile in this case could be searched using the ATS to guarantee success; however, the cost in terms of computational resources would be excessive. The numbers of partitions and components could be changed according to the complexity of the profile. This strategy for parameter adjustments is beneficial to balance the alignment performance, time, and costs. From a methodological point of view, additional target components are helpful in correcting the nonlinear retention time shift. The ATS method is an automated method with several parameters. Thus, it is effective in the analysis of high-throughput profiles. The study of metabonomic mixtures with hundreds or more sample profiles obtained from autosampling, high-dimensional instruments is universal. The conventional methods have limitations with respect to the treatment of such data sets. In addition, the prior selection of components with a maximum response for alignment also dramatically improves the performance by neglecting the possible influences of small components in some cases; it also substantially reduces the time-consuming computations.

Most parameters of the ATS method and the entire shift correction shown in Figure 2 were not particularly sensitive to the final alignment results, which made the proposed method more powerful in treating complex metabolite profiles with hundreds or more small molecules. However, the complicated profiles should be analyzed carefully because of the large background shift interferences if no previous (semi)automatic data pretreatment is used; thus, the determination of parameters should be conservative to reduce the effect on searching the target components. A prestudy may be useful in the selection of appropriate parameters for experimental data, such as the determination of a possible base shift and heteroscedastic noise using chemometric methods.

## 4. Conclusions

The automated or semiautomated alignment of the retention time shift is a primary focus in biomarker discovery, metabolomics, and systems biology research. In this study, a new method was developed that focuses on the correction of high-dimensional and high-throughput metabolite profiles, including the selection of a standard profile with information content, the determination of target components as a reference for alignment using the ATS method, and the development of an effective data piecewise partition strategy for global searching. The exact elution information of target components could be automatically acquired by the ATS method. With this information, the LIT technique can be further used to complete the entire correction among the standard and sample profiles. The strategy of piecewise partition for the standard profile also significantly improved the ability to recognize the target components and the efficiency of the search. This type of automatic analysis can be convenient for the treatment of high-dimensional and high-throughput profiles, which is important to improve the performance of subsequent data treatment, such as pattern recognition, the identification similarities and differences among metabolite profiles or fingerprinting, and the deconvolution of complex peaks, in studies related to life sciences.

## Symbols and Annotations

*φ*_*i*_: Information content (IC)
**x**_*i*_: The *i*th TIC metabolite profile
*N*: The number of the parts that the standard profile was uniformly partitioned
*g*_*g*_: The number of peak clusters recorded to find the target component in aligned chromatogram within each partition
*c*_*c*_: The number of division in each partition
**X**: Data set applied for correction as standard reference
**Y**_*m*×*n*_: Data set with *m* chromatographic scan points and *n* spectrometric scan points
**U**: Score matrix after SVD analysis of data **X**
**V**: Loading matrix after SVD analysis of data **X**
**S**: A diagonal matrix that collects the square root of all of the eigenvalues of data set **X**
**V**_*p*_: The first *p* eigenvectors of the abstract matrix spectral** V**
**O**_*P*_: An orthogonal projection matrix
**y**_*i*_: Every column vector of **Y**, the spectrum obtained from the *i*th chromatographic point *i*
**y**_*i*_^resi^: The residual vector containing the remaining information after **Y** at the *i*th chromatographic point projecting to **X**
**ζ**_*i*_: The congruence coefficient (inner product) between the original spectrum **y**_*i*_ and the projected residual vector **y**_*i*_^resi^.
